# Turning the knobs: The impact of glutathionylation on starch metabolism

**DOI:** 10.1093/plphys/kiaf322

**Published:** 2025-07-23

**Authors:** Anna Moseler

**Affiliations:** Assistant Features Editor, Plant Physiology, American Society of Plant Biologists, Rockville, MD 20855, USA; INRES-Chemical Signalling, University of Bonn, Bonn 53117, Germany

Stomata, tiny pores found on the surface of leaves and other plant organs, play a crucial role in regulating gas exchange and transpiration. They typically consist of two bean- or dumbbell-shaped guard cells that flank a central opening in the impermeable waxy cuticle (Sussmilch et al. 2019). Most plants can actively open and close stomata in response to endogenous and environmental signals. The regulation of stomatal movement is highly complex and dependent on numerous triggers, including light, phytohormones, Ca^2+^, H_2_O_2_ (hydrogen peroxide), and changes in metabolite levels, such as starch (Flütsch et al. 2020; Schulze et al. 2021; Rodrigues and Shan 2022; Bahadar et al. 2025). In Arabidopsis guard cells, energy-storing starch is rapidly degraded to glucose at dawn. This starch breakdown is catalyzed by the glucan hydrolases α-AMYLASE3 (AMY3) and β-AMYLASE1 (BAM1) and promotes stomatal opening to achieve efficient photosynthesis (Flütsch et al. 2020). Both enzymes, AMY3 and BAM1, previously were shown to be redox-regulated through the formation of an inhibitory intramolecular disulfide bond (Sparla et al. 2006; Seung et al. 2013). AMY3 can be further inactivated by glutathionylation, a reversible post-translational modification in which a glutathione molecule is covalently attached to a cysteine thiol (-SH) group to form a protein-glutathione mixed disulfide (Gurrieri et al. 2019). Proteins can be glutathionylated via different pathways, including enzymatic processes that involve class I glutaredoxins (GRXs), or non-enzymatic processes that involve reactions of reduced glutathione (GSH) with sulfenic acids (-SOH) or of oxidized glutathione (GSSG) with reduced thiols. Notably, the glutathione pool in cell compartments containing a glutathione reductase (GR) is highly reduced (≥50,000:1 GSH:GSSG), resulting in low-millimolar concentrations of GSH and only nanomolar concentrations of GSSG (Müller-Schüssele et al. 2021; Bohle et al. 2024). GR efficiently regenerates two GSH from GSSG, and a decrease of GR activity leads to increased GSSG levels (Yu et al. 2013; Marty et al. 2019). How (sub)cellular glutathione homeostasis is linked with redox-mediated regulation of starch metabolism and concomitant stomata opening has so far been unclear.

In the present study, Gurrieri et al. (2025) revealed a protective function of glutathionylation on starch-degrading BAM1 activity in vitro. Additionally, they showed that the glutathione redox state in plastids impacts starch metabolism and stomatal movement.

In an initial experiment, the authors demonstrated that oxidative conditions inactivate BAM1 through the formation of an intramolecular disulfide bridge. This inactivation was reversed under reducing conditions, indicating that the reduction of the disulfide bridge restores BAM1 activity. Presence of GSH slowed down H_2_O_2_-induced inhibition of BAM1, suggesting that BAM1 glutathionylation limits the formation of the inhibitory disulfide. It is worth mentioning that GSSG formation through the reaction of H_2_O_2_ with GSH is very slow (Smirnoff and Arnaud 2019), which means that after H_2_O_2_ + GSH treatment, BAM1 glutathionylation occurs by the reaction of sulfenic acid with GSH rather than GSSG with the reduced thiol. Western blots with anti-GSH antibodies confirmed BAM1 glutathionylation after incubation with either H_2_O_2_ + GSH or with oxidized GSSG as control to disentangle the direct effect of H_2_O_2_ from those of glutathionylation. Because BAM1 harbors 8 cysteines, the authors further showed that the glutathionylation resulting from both treatments targeted the same cysteine residue. While BAM1 activity was decreased in presence of H_2_O_2_ + GSH, GSSG did not inhibit BAM1, ruling out that the enzymatic inactivation is a result from glutathionylation but rather of disulfide bond formation through H_2_O_2_. The reversibility of BAM1 glutathionylation was tested using the plastidic glutaredoxins GRXC5 and GRXS12 as well as GSH and the reductant dithiothreitol (DTT). Both GRXs and DTT effectively removed the glutathione modifications, while GSH alone was less efficient, pointing to a regulatory role of the plastidic GRXs in BAM1 de-glutathionylation.

To further study how glutathionylation affects BAM1 activity, Gurrieri and colleagues measured BAM1 activity after incubating BAM1 with glutathionylating agents (H_2_O_2_ + GSH or GSSG) and subsequent desalting to remove excess reagents. The glutathionylation on BAM1 was spontaneously lost over time, as deduced from the release of free GSH, and samples that lost the glutathionylation signal showed the lowest BAM1 activity. After the glutathionylation-mediated inhibition of BAM1, the authors tested if the plastidic proteins thioredoxin f1 (TRXf1), GRXC5, or GRXS12 are able to restore BAM1 activity. While GRXC5 and GRXS12 were unable to restore BAM1 activity efficiently, TRXf1 led to a BAM1 reactivation. In summary, these in vitro data show that H_2_O_2_ inhibits BAM1 activity by promoting the formation of a reversible intramolecular disulfide bond, which can be temporarily prevented by BAM1 glutathionylation. Over time, this glutathionylation is spontaneously lost, leading to disulfide formation and enzyme inhibition, which can be efficiently reversed by TRXf1 (Figure.).

**Figure.**
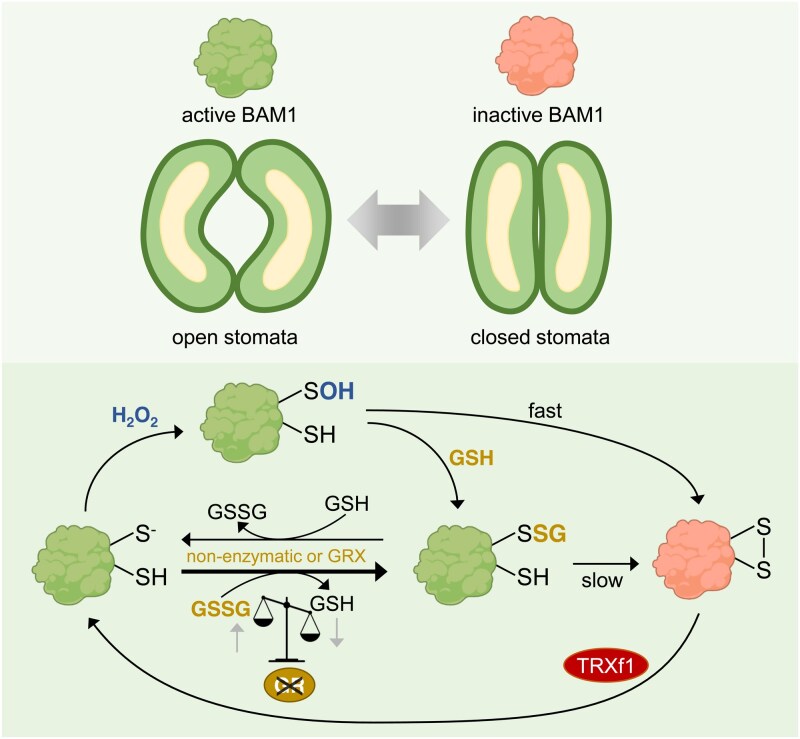
Model of BAM1 redox regulation. The glucan hydrolase BAM1 is needed to break down starch in guard cell chloroplasts and to promote stomata opening. BAM1 gets sulfenylated (-SOH) by reacting with H_2_O_2_. The sulfenylation is either removed by forming an intramolecular disulfide bond with a cysteine from the active site, which inactivates BAM1, or by reacting with GSH, leading to glutathionylation (-SSG) and maintaining the enzyme's activity. Glutathionylation can occur also by direct reaction with GSSG, the amount of which increases when glutathione reductase is not active. Glutathionylation can be removed by the active site cysteine, leading to an intramolecular disulfide bond and inhibition of activity. Depending on the glutathione homeostasis, GRXs can catalyze glutathionylation or de-glutathionylation of proteins. The inhibitory intramolecular disulfide bond of BAM1 can be reduced by plastidic thioredoxin TRXf1 (modified after Gurrieri et al. (2025)).

In the next step, Gurrieri et al. explored the impact of plastidic glutathione homeostasis on BAM1 activity in planta. They investigated protein levels and activity of BAM1 in two Arabidopsis mutants that have reduced activity of GR2, a glutathione reductase that is dual-targeted to plastids and mitochondria. The *miao* mutant has minimal GR2 activity and the *epc-2* mutant, which is a *gr2* null mutant complemented with a plastid-localized GR2, has ∼25% of wild-type GR2 activity (Yu et al. 2013; Marty et al. 2019). Since BAM1 is mainly expressed in guard cells during early development, epidermal peels were sampled at specific time points over a dark-to-light transition, when starch degradation and BAM1 activity are most relevant. Western-blot analysis showed that BAM1 protein levels were slightly lower in both GR2-deficient mutants compared with wild-type control plants. Native gel analysis revealed that in wild-type plants, BAM1 activity increased after DTT treatment, indicating redox regulation. In contrast, *miao* and *epc-2* mutants showed high BAM1 activity even without DTT treatment, and no further activation was observed upon DTT addition. In line with the increased BAM1 activity in plants with diminished GR2 activity, the starch content of *miao* and *epc-2* plants was lower than that of wild-type plants, while *bam1* knock-out plants showed elevated starch content. These data indicate that a decrease in the plastidic [GSH]/[GSSG] ratio keeps BAM1 glutathionylated, and thus BAM1 stays active for a longer time and thus lowers the starch content. To understand the consequences of altered glutathione homeostasis and starch content on stomata aperture, the team measured the stomatal behavior of the respective mutants. Unlike *bam1* mutants, which tend to keep their stomata more closed in the light—likely due to decreased starch breakdown—*epc-2* mutants maintain relatively open stomata even in the dark. This matches their low starch levels in guard cells. Comparing *bam1* and *epc-2* mutants revealed a clear inverse relationship between starch content (linked to BAM1 activity) and stomatal opening. Such an inverse relationship, however, was not seen in *miao* mutants, which was not further investigated by the authors.

In summary, Gurrieri et al. illustrate how glutathionylation can serve as a transiently protective post-translational modification by interfering with the formation of inhibitory disulfide bonds in BAM1. The regulation of BAM1 by TRXf1 and glutathione provides a sophisticated mechanism to control starch degradation in guard cells and stomatal behavior. Further studies are needed to unravel which cysteine of BAM1 are actually targeted and how redox control happens in response to endogenous and environmental signals. Additionally, *epc-2* is useful in studying the contribution of mitochondrial GR to the overall phenotype of the mutant. A more general question to address is how the redox regulation interacts with other triggers such as phytohormones and what role neighboring cells, both epidermal and mesophyll, play in stomata movement. The insights of this study, however, pave the way to further study redox-mediated regulation of metabolic rearrangements and their impact on stomata opening. Thus, this work could set the ground to mechanistically understand important crop traits such as stomatal efficiency and drought tolerance.

## Data Availability

No new data included in this article.
